# Dose-response effects of acute exercise intensity on state anxiety among women with depression

**DOI:** 10.3389/fpsyt.2023.1090077

**Published:** 2023-05-12

**Authors:** Seana L. Perkins, Dane B. Cook, Matthew P. Herring, Jacob D. Meyer

**Affiliations:** ^1^Department of Kinesiology, Iowa State University, Ames, IA, United States; ^2^Department of Kinesiology, University of Wisconsin, Madison, WI, United States; ^3^Department of Physical Education and Sport Sciences, University of Limerick, Limerick, Ireland

**Keywords:** anxiety, depression, aerobic exercise, comorbid, moderate intensity

## Abstract

Anxiety is common in people with major depressive disorder (MDD), yet the anxiolytic effects of acute exercise in MDD are unknown. The purpose of this analysis was to determine a potentially optimal acute exercise intensity for reducing state anxiety in women with MDD, the duration of the response, and the potential influences of depression severity and preferred-intensity exercise. Using a within-subject, randomized, counter-balanced design, 24 participants completed five separate visits including 20  min of steady-state bicycling at prescribed (*via* RPE) light, moderate, or hard intensities, a preferred/self-selected session, or a quiet rest (QR) session. State anxiety was measured *via* the State–Trait Anxiety Inventory (STAI-Y1) and anxiety visual analog scale (VAS) at pre-, immediately (VAS only), 10  min, and 30  min post-exercise. Depression was measured *via* the Beck Depression Inventory (BDI-II) pre-exercise. Moderate exercise resulted in a moderate state anxiety reduction compared to QR 10 min (STAI-Y1: *g* = 0.59, *p*_adj_ = 0.040) and 30 min post-exercise (STAI-Y1: *g* = 0.61, *p*_adj_ = 0.032). Pairwise differences indicated each exercise session decreased state anxiety pre to 10 min and 30 min post-exercise (all *p*_adj_ < 0.05) for the STAI-Y1, and for moderate and hard exercise from pre to each time point post-exercise (all *p*_adj_ < 0.05) for the VAS. Depression severity was associated with state anxiety (*p* < 0.01) but did not influence the overall results. Prescribed moderate intensity exercise led to greater reductions in state anxiety compared to preferred at 30 m (STAI-Y1: *g* = 0.43, *p* = 0.04). These findings suggest steady-state prescribed moderate exercise reduces state anxiety in women with MDD for at least 30 min following exercise regardless of their depression severity.

## 1. Introduction

Depression and anxiety are prevalent, highly comorbid, and poorly treated public health concerns associated with disability, high healthcare utilization, and low quality of life (1–3). In 2019, depression affected over 10% of US adults, with females affected at twice the rate of males, while Generalized Anxiety Disorder (GAD) affected 19% of females and almost 12% of males (4, 5). With high rates of anxiety and depression comorbidity, identifying and optimizing treatment strategies that influence both depression and anxiety is needed.

Chronic and acute exercise reduce symptoms of both depression and anxiety (6–9). Chronic exercise training can treat depression and anxiety through a multitude of potential mechanisms including repeated exposure to the acute beneficial effects of each individual exercise session. Acute exercise has immediate anxiolytic effects in healthy and clinical populations (7, 10); however, it is not clear if acute exercise reduces state anxiety in adults with depression. Further, the antidepressant effects of aerobic exercise can be inhibited by high anxiety (11). It is possible that the inverse is true—depression severity may blunt the anxiolytic effects of exercise; however, this is unknown. Further research may lead to a better understanding of how anxiety and depression severity influence the beneficial effect of exercise, leading to improved exercise-based treatments for people with depression.

The exact influence of features of the exercise stimulus, particularly the exercise intensity, on the anxiety response remain unclear. While more exercise is often better, with vigorous exercise providing roughly double the benefit of moderate exercise in the US Physical Activity Guidelines (12), there may be a different dose–response relationship between exercise intensity and the mood state response. Generally, vigorous exercise appears to acutely reduce state anxiety (7, 10) and anxiety sensitivity (13). Yet, immediately after vigorous exercise, state anxiety may *increase* briefly (14) and during steady-state vigorous exercise there is often a negative affective experience (15). This anxiogenic vigorous exercise experience could be due to the similarity of somatic symptoms of anxiety and the cardiopulmonary response to vigorous exercise (i.e., increased heart rate, breathing rate, etc.), leading to interpreting the physiological response to intense exercise as anxiety symptoms. Therefore, we hypothesized that moderate, rather than vigorous, exercise would provide an optimal stimulus for reducing state anxiety in women with major depressive disorder (MDD). Due to the physiological recovery from exercise and the potential increase in state anxiety following exercise, we hypothesize that the state anxiety reduction will strengthen following exercise cessation. However, it is possible that the acute response is at its highest immediately post-exercise and decays over time, particularly if it is tied to during-exercise physiological changes. Therefore, evaluating the time course of changes, particularly across different exercise intensities with their distinct physiological demands, is warranted.

We previously examined the acute effects of sessions of prescribed light, moderate, and hard exercise compared to quiet rest on depressed mood state in female adults with MDD (16). All three intensities significantly improved depressed mood state up to 30 min after exercise. However, due to the commonality and impact of anxiety symptoms in depressed populations, the study reported here augments and extends this prior work by evaluating the dose–response effects of acute exercise intensity on state anxiety in a depressed population. As anxiety may limit exercise-induced depressive symptom relief (11), we also consider depression severity as a potential confounder of the dose–response relationship. Lastly, exercise at a self-selected intensity may be more beneficial than prescribed exercise (17), potentially due to higher autonomy associated with self-selecting the intensity. While the current literature shows that exercise can be a useful acute anxiolytic in healthy populations, understanding the roles of intensity, depression severity, and self-selection of the intensity impact the acute state anxiety response in depressed adults are important next steps in determining optimal exercise prescriptions for adults with MDD.

Therefore, this analysis (1) investigates the magnitude and duration of state anxiety effects of light, moderate, and hard prescribed steady-state exercise intensities compared to quiet rest, (2) assesses the influence of depressive symptom severity on the acute state anxiety response, and (3) compares the state anxiety effects of a preferred exercise session to the prescribed sessions and quiet rest. We hypothesized that (1a) moderate intensity exercise would result in the strongest decrease in state anxiety, (1b) the anxiolytic response would strengthen over time, (2) depressive symptom severity would inhibit the state anxiety response to exercise, and (3) preferred exercise would elicit stronger anxiolytic effects than any other session.

## 2. Methods

These data come from a randomized, counter-balanced within-subject study evaluating the effects of acute exercise intensity on depressed mood state in women with MDD. Full methodological details have previously been published (16, 18). The following methods are limited to those that pertain to this analysis focused on the state anxiety response to exercise.

### 2.1. Participants

Twenty-four women diagnosed with MDD completed the study. Inclusion criteria were being female, between the ages of 20 and 60, diagnosed with MDD via a structured clinical interview (Mini International Neuropsychiatric Interview 6.0.0; MINI), no medical contraindications to safe participation in exercise (Physical Activity Readiness Questionnaire; PAR-Q), and on a stable psychiatric treatment regimen for at least 8 weeks prior to the baseline visit. Exclusion criteria were evaluated based on participants’ self-report and included being or planning to become pregnant, being a current smoker, being diagnosed with any comorbid psychological disorders [other than GAD due to the high comorbidity, assessed via the MINI; (1)], currently taking opioids or analgesic medications, or currently abusing alcohol or other drugs (identified via MINI).

### 2.2. Procedures

Five visits including sessions of quiet rest, light, moderate, and hard steady-state exercise, and a preferred-intensity condition were completed by each participant, with sessions separated by at least one week. The initial visit consisted of informed consent procedures, completing a structured clinical interview (MINI) to verify eligibility, self-reporting demographic details, completing baseline questionnaires, and performing one of the five sessions. On the four subsequent visits, each participant completed the exact same procedures as the first visit without the informed consent, demographic questionnaires, and MINI.

For each session, participants completed the International Physical Activity Questionnaire (IPAQ), Beck Depression Inventory (BDI-II), and the State–Trait Anxiety Inventory (STAI-Y1; State version only) and anxiety visual analog scale (VAS) to record current state anxiety. Next, participants completed one of five randomized and counterbalanced experimental sessions (described below). Immediately after completion of the assigned session, participants completed the anxiety VAS. The STAI-Y1 and VAS were completed again at 10- and 30-min post-session for a total of 3 completions for the STAI-Y1 (pre, 10 m, and 30 m post) and 4 completions for the VAS (pre, immediately post, 10 m, and 30 m post). All procedures were approved by the local Institutional Review Board and informed consent was obtained from each participant.

#### 2.2.1. Sessions

Exercise sessions included a light, moderate, hard, and preferred intensity 30-min cycling session. Prescribed intensities were prescribed at ratings of perceived exertion [RPE; (19)] of “11,” “13,” and “15,” respectively, and preferred intensity sessions were at any intensity participants chose [see (18) for further detail]. All sessions were completed on an electronically braked cycle ergometer (Lode Corival, Lode BV, Groningen, The Netherlands). Participants were instructed to begin with a 5-min warm-up pedaling at 60–70 rpm and gradually increase their exertion through modulation of the resistance to reach the assigned or their preferred intensity at the 5-min mark. Next, participants completed 20 min of exercise and maintained 60–70 rpm. They were instructed to adjust the resistance as needed to maintain the prescribed RPE for prescribed sessions. For the preferred session, participants were free to adjust their intensity throughout the session with no external guidance on how to conduct the exercise aside from maintaining the same cadence as in the other sessions (60–70 rpm). The last 5 min of all sessions consisted of a cool-down period in which resistance was reduced to 0 Watts and participants could pedal at any speed, totaling 30 min of cycling. Perceived exertion was queried every 5 mins during the steady-state period and, if RPE was not at the prescribed intensity (prescribed sessions only), participants were encouraged to alter the resistance as needed to achieve the prescribed rating (11, 13, or 15) or adjusted whenever the participant wanted (preferred session).

#### 2.2.2. Physiological measurement

Physiological measurements were obtained and recorded to verify exercise intensity. Resistance (Watts) from the cycle ergometer was recorded every time the participant made a change. Physiological measurements (ventilation, oxygen consumption, carbon dioxide production) were obtained continuously and analyzed with 15-s averaging using a metabolic cart (TrueOne, ParvoMedics, Sandy, UT) and two-way non-rebreathing valve (Hans-Rudolph, Kansas City, MO). Heart rate was measured and recorded throughout exercise and recovery via chest-worn heart rate monitor (Polar, Lake Success, NY). Percent of maximum heart rate was calculated using participants’ mean age in the age-predicted maximum heart rate equation (20).

### 2.3. Questionnaires

Participants self-reported age, height and weight, and current medication usage. The Physical Activity Readiness Questionnaire [PAR-Q; (21)] was used to verify safety to exercise (scores > 0 indicate it is unsafe to exercise). No participants in the present study scored above a “0.”

The International Physical Activity Questionnaire [IPAQ; (22)] is a self-report questionnaire that asks the participant to recall physical activity over the previous 7 days. The IPAQ is a well-established questionnaire that has comparable criterion validity to other self-report activity measures [*ρ* = 0.3; (22)]. A research staff member conducted this questionnaire verbally.

Clinical mental health diagnoses and depression severity were assessed using the MINI and BDI-II. The Mini International Neuropsychiatric Interview 6.0.0 [MINI; (23)] is a validated mental health interview used to diagnose DSM-IV mood disorders (23–25). This was used to assess current depressive episode and to evaluate the presence of any other exclusionary diagnoses. The Beck Depression Inventory-II [BDI-II; (26)] was used to assess current depressive symptoms. The BDI-II is a validated measure for measuring level of depression and has good reliability [Cronbach’s α = 0.91; (26)]. Data from the present sample showed good internal consistency for the BDI-II (Cronbach’s α = 0.89).

State anxiety was assessed using the STAI-Y1 and anxiety visual analog scale (VAS). The State–Trait Anxiety Inventory [STAI-Y1; (27)] consists of 20 statements on a 4-point Likert-like scale and assesses current state anxiety. The STAI-Y1 has demonstrated good criterion validity for measuring state anxiety when compared to other established anxiety measures [*r* = 0.73–0.85; (28)]. For adults, scores of 39–40 have been suggested to detect clinically significant symptoms for the state subscale (29). The anxiety visual analog scale (VAS) used in the present study was a 10 cm-long line on which participants marked their current anxiety which was scored 0 to 100 (“No anxiety at all” to “Worst anxiety imaginable”) with participants answering based on how they felt in that moment.

### 2.4. Data analysis

All data were analyzed using R version 4.0.1 or higher (R Core Team, 2020). The level of significance (α) was set to 0.05 for all main effects and interaction analyses. A sensitivity analysis (using G^*^Power 3.1.9.6) was conducted to determine the size of the effect that could be identified using the present design. With an alpha of 0.05, *β* of 0.8, the correlations among repeated measures for each dependent measure (*r* = 0.768 and 0.823 for primary STAI and VAS models, respectively), and nonsphericity corrections for each model (epsilon = 0.611 and 0.472, respectively), a *d*-effect size of at least 0.475 (VAS) and up to 0.542 (STAI) was needed to detect a significant interaction. Participant characteristics were evaluated descriptively with counts, means and standard deviations.

Aim #1 was evaluated with both the STAI-Y1 and VAS. To evaluate this, a three time (pre, 10 m, 30 m) by four session (quiet rest, mild, moderate, and hard) repeated-measures ANOVA was conducted with STAI-Y1 as the dependent variable. Session was defined using prescribed RPE categories due to its ease and efficiency, translational practicality, and internally valid approach to describing sessions, as each participant completed one session at each intensity. Similarly, a four time (pre, post, 10 m, 30 m) by four session (quiet rest, light, moderate, hard) repeated-measures ANOVA was conducted with the anxiety VAS as the dependent variable. The Greenhouse–Geisser correction was used when the sphericity assumption was violated due to ε values below 0.75. Pairwise comparisons evaluated differences across time and between sessions using Bonferroni corrections for multiple comparisons. Hedges’ *g* (30) was used to quantify the magnitude of differences for each aim. As the order of the visits may have influenced the anxiety response to each session, we have explored the potential for visit (i.e., first, second, third, fourth, or fifth) to interact by including it in additional exploratory STAI and VAS models. Two participants had missing VAS data for their first visit and were not included in ANOVA analyses, but all other data were present across all sessions and visits.

To assess the association of current depressive symptom severity on the acute anxiolytic response (aim #2), both the STAI-Y1 and VAS models were repeated including BDI total score as a covariate. Simple correlations between BDI-II score and changes in STAI-Y1 at 10 and 30 min were also calculated overall and for each session separately using Spearman’s rho.

To assess the effectiveness of a preferred exercise session against prescribed sessions and compared to quiet rest (aim #3), the STAI-Y1 and VAS models in aim #1 were repeated, with the addition of the preferred session.

## 3. Results

Participant characteristics are presented in Table 1. All 24 participants had a MDD diagnosis and 5 also had a GAD diagnosis. Fourteen participants were taking antidepressants and 3 taking anxiety medication (two taking Clonazepam and one taking Buspirone). At baseline, the average STAI-Y1 (full sample mean = 48.2; GAD only = 51.6) suggest clinical significance and BDI-II scores (mean = 26.3) were of moderate severity according to standard cutoffs (29, 31). Changes in STAI scores (pre-10 min, pre-30 min, and 10–30 min) for each session were not significantly different between those with GAD and those without; therefore, all analyses are based on the whole sample. The supplementary analyses evaluating the potential influence of visit order (i.e., first, second, third, fourth, or fifth session) on the STAI and VAS models indicated no significant session-by-time-by-visit interaction nor a main effect of visit when included as an independent predictor. Therefore, the following model results are from models that do not include visit.

**Table 1:** Sample characteristics (*n* = 24).

Measure	Mean ± SD or *n*
Age (yrs)	38.6 ± 14
BMI (kg/m^2^)	29.7 ± 8.0
MVPA (min/day)	77.1 ± 82.9
MDD Diagnosis (*n*)	24
GAD Diagnosis (*n*)	5
Baseline STAI-Y1 Score	48.2 ± 8.6
Baseline BDI-II Score^a^	26.3 ± 8.2
Taking Antidepressants (*n*)	14
No Antidepressants (*n*)	10
Taking Anxiety Medication (*n*)	3

Values are mean ± standard deviation unless otherwise specified. ^a^Beck Depression Inventory (BDI-II) scores indicate mild to severe depression. BDI-II, Beck Depression Inventory; BMI, Body Mass Index; MDD, Major Depressive Disorder; GAD, Generalized Anxiety Disorder; MVPA, Moderate to Vigorous Physical Activity; SD, Standard Deviation; STAI-Y1, State–Trait Anxiety Inventory (State version only).

### 3.1. Session manipulation

Overall averages of RPE for the quiet rest, light, moderate, and hard sessions (prescribed at RPE = 6, 11, 13, and 15, respectively) were 6.2 ± 0.5, 10.9 ± 0.3, 13.2 ± 0.5, and 15.1 ± 0.2, respectively, indicating successful manipulation of intensity across the sessions. Mean RPE for the preferred session was 12.5 ± 1.7 (moderate) and ranged across participants from 8.5 (very light) to 16 (very hard). Mean resistance (Watts) values recorded across each prescribed 20-min session were 0.0 ± 0.0, 45.6 ± 23.8, 72.8 ± 32.8, and 93.0 ± 34.3, respectively, and 70.7 ± 33.2 for the preferred session. Mean heart rate (bpm) and percent of maximum heart rate measured during each prescribed session were 82.9 ± 11.1 (45.8%), 113.8 ± 20.8 (62.9%), 130.8 ± 22.1 (72.3%), and 149.4 ± 22.9 (82.6%), respectively, and 139.3 ± 45.1 for the preferred session. As originally reported by Meyer et al. [(16); see Table 2], prescribed exercise intensities were statistically significant from each other for each measure of intensity.

**Table 2:** Mean STAI-Y1 scores and effect sizes before and after 30 min of exercise.

Session	STAI-Y1			Session effect sizes session vs. quiet rest			Time effect sizes within session changes		
	Pre	10 m	30 m	Pre	10 m	30 m	Pre – 10 m	Pre – 30 m	10 m – 30 m
Quiet Rest	48.9 ± 7.1	44.3 ± 7.3	43.7 ± 7.6	–	–	–	**1.01 (0.74, 1.42)** ^**^	**0.97 (0.71. 1.4)** ^**^	0.17 (−0.29, 0.51)
Light	46.8 ± 7.8	41.9 ± 8.8	40.5 ± 10.1	0.33 (−0.05, 0.78)	0.32 (−0.04, 0.77)	0.32 (−0.06, 0.93)	**0.84 (0.57, 1.25)** ^**^	**0.77 (0.42, 1.17)** ^**^	0.31 (−0.06, 0.65)
Moderate	48.2 ± 9.8	40.5 ± 7.5	39.1 ± 9.3	0.08 (−0.31, 0.50)	**0.59 (0.20, 1.14)** ^*^	**0.61 (0.23, 1.19)** ^*^	**1.09 (0.75, 1.71)** ^**^	**1.01 (0.73, 1.47)** ^**^	0.28 (−0.10, 0.75)
Hard	46.1 ± 8.4	40.6 ± 10.3	38.9 ± 11.4	0.32 (−0.07, 0.84)	0.38 (−0.02, 0.95)	**0.51 (0.11, 0.98)** ^*^	**0.70 (0.37, 1.05)** ^**^	**0.94 (0.65, 1.35)** ^**^	**0.43 (0.01, 0.91)** ^*^
Preferred	45.8 ± 8.8	42.3 ± 9.1	41.6 ± 9.2	0.33 (−0.08, 0.96)	0.24 (−0.14, 0.82)	0.25 (−0.14, 0.67)	**0.55 (0.20, 1.06)** ^**^	**0.52 (0.19, 1.02)** ^**^	0.15 (−0.30, 0.53)

Mean State–Trait Anxiety Inventory (STAI-Y1) ± standard deviation and effect sizes (Hedges’ g [95% Confidence Interval]) comparing average STAI responses to each exercise session to responses to quiet rest at 10 and 30 min post-exercise and compared across time within each session. ^*^Indicates a significant pairwise difference (*p* < 0.05) before Bonferroni correction. ^**^Indicates a significant pairwise difference (*p* < 0.05) after Bonferroni correction.

### 3.2. Hypothesis #1a: dose response comparisons between prescribed intensities across time

#### 3.2.1. STAI-Y1 results

There were significant main effects of time (*F*_(1.34,30.73)_ = 40.37, *p* < 0.001, ε = 0.668) and session (*F*_(3,69)=_3.51, *p* = 0.02, ε = 0.809), but no significant session by time interaction (*F*_(3.66,84.26)_ = 1.44, *p* = 0.23, ε = 0.611). Pairwise comparisons showed moderate intensity exercise resulted in a significant moderate anxiety reduction compared to quiet rest at 10 m (*t* = 2.98, *g* = 0.59, *p*_adj_ = 0.040; Table 2) and 30 m post-exercise (*t* = 3.08, *g* = 0.61, *p*_adj_ = 0.032) after Bonferroni correction. No other pairwise comparisons were significant. While hard intensity resulted in a moderately lower anxiety score (*t* = 2.58, *g* = 0.51, *p* = 0.017) at 30 min post-exercise compared to quiet rest, the comparison was not significant after correction (*p*_adj_ = 0.101).

**Table 3:** Mean anxiety VAS scores and effect sizes before and after 30 min of exercise.

	Anxiety VAS				Session effect sizes session vs. quiet rest				Time effect sizes within session changes					
Session	Pre	Post	10 m	30 m	Pre	Post	10 m	30 m	Pre–Post	Pre—10 m	Pre—30 m	Post—10 m	Post—30 m	10 m–30 m
Quiet Rest	38.8 ± 20.5	33.7 ± 20.4	31.3 ± 19.7	29.6 ± 20.5	–	–	–	–	0.37 (0.01, 0.75)	**0.66 (0.37, 1.05)** ^**^	**0.73 (0.39, 1.20)** ^**^	0.30 (−0.07, 0.77)	**0.43 (0.04, 0.97)** ^*^	0.28 (−0.12, 0.66)
Light	36.1 ± 28.9	27.7 ± 20.8	30.5 ± 26.6	27.8 ± 26.4	0.09 (−0.27, 0.72)	0.31 (−0.08, 0.77)	0.03 (−0.33, 0.69)	0.06 (−0.29, 0.63)	0.39 (−0.01, 0.78)	0.29 (−0.12, 0.88)	**0.47 (0.07, 0.89)** ^*^	0.19 (−0.23, 0.55)	0.01 (−0.48, 0.36)	0.37 (0.00, 0.67)
Moderate	41.1 ± 24.5	24.6 ± 20.2	24.1 ± 20.2	24.7 ± 20.0	0.11 (−0.33, 0.47)	**0.45 (0.08, 0.87)** ^*^	**0.48 (0.11, 0.97)** ^*^	0.30 (−0.11, 0.81)	**1.20 (0.83, 1.83)** ^**^	**0.93 (0.66, 1.32)** ^**^	**0.80 (0.50, 1.23)** ^**^	0.04 (−0.36, 0.49)	0.01 (−0.43, 0.42)	0.08 (−0.32, 0.50)
Hard	40.5 ± 25.3	28.9 ± 24.0	25.5 ± 22.9	25.7 ± 23.5	0.02 (−0.42, 0.44)	0.25 (−0.12, 0.68)	0.31 (−0.06, 0.88)	0.20 (−0.18, 0.74)	**0.71 (0.36, 1.16)** ^**^	**0.88 (0.59, 1.33)** ^**^	**0.85 (0.61, 1.25)** ^**^	**0.46 (0.10, 0.95)** ^*^	0.39 (−0.03, 0.92)	0.03 (−0.45, 0.42)
Preferred	38.1 ± 24.6	27.9 ± 22.6	26.8 ± 22.8	27.8 ± 23.0	0.03 (−0.36, 0.43)	0.29 (−0.08, 0.77)	0.26 (−0.13, 0.89)	0.09 (−0.31, 0.49)	**0.67 (0.32, 1.1)** ^**^	**0.69 (0.36, 1.16)** ^**^	**0.49 (0.13, 0.89)** ^*^	0.10 (−0.26, 0.54)	0.01 (−0.39, 0.44)	0.12 (−0.28, 0.51)

Mean Anxiety Visual Analog Scale (VAS) scores ± standard deviation and effect sizes (Hedges’ g [95% Confidence Interval]) comparing average VAS responses to each exercise session to responses to quiet rest at immediately (Post), 10 and 30 min post-exercise and compared across time within each session. ^*^Indicates a significant pairwise difference (*p* < 0.05) before Bonferroni correction. ^**^Indicates a significant pairwise difference (*p* < 0.05) after Bonferroni correction.

#### 3.2.2. VAS results

State anxiety scores from the VAS showed a main effect of time (*F*_(1.79,37.63)_ = 22.32, *p* < 0.001, ε = 0.597), but no significant session by time interaction (*F*_(4.25,89.15)_ = 1.598, *p* = 0.18, ε = 0.472) or main effect of session (*F*_(3,63)_ = 0.432, *p* = 0.73, ε = 0.833). Pairwise comparisons indicated that moderate intensity significantly reduced anxiety symptom severity immediately post-exercise (*t* = 2.26, *g* = 0.45, *p* = 0.034; Table 3) and 10 m post-exercise (*t* = 2.41, *g* = 0.48, *p* = 0.024) compared to quiet rest, though neither pairwise comparison was significant after Bonferroni correction (all *p* > 0.05).

### 3.3. Hypothesis #1b: effects across time within session

For the STAI-Y1 and after Bonferroni correction, significant pairwise differences were found for each session (quiet rest, light, moderate, and hard) strengthening and improving from pre to 10 m and 30 m post-exercise (all *p*_adj_ < 0.05), with no differences between 10 m and 30 m post-exercise for any session (all *p*_adj_ > 0.05). For the VAS and after Bonferroni correction, significant pairwise differences were found for quiet rest from pre to 10 m and 30 m post-exercise, for moderate intensity from pre to post, 10 m, and 30 m post-exercise, and for hard intensity from pre to post, 10 m, and 30 m post-exercise (all *p*_adj_ < 0.05), all showing improvements in anxiety symptoms. There were no significant differences across time for light intensity (all *p*_adj_ > 0.05).

### 3.4. Hypothesis #2: influence of depressive symptoms on anxiety relief

When added to the STAI-Y1 model, BDI was significantly associated with state anxiety (*p* < 0.01). The significance of the session by time interaction and main effect of time did not change, but session became nonsignificant (*p* = 0.0502) when adjusting for total depressive symptom score. Spearman’s rho correlation coefficients were only significant between BDI and changes in STAI-Y1 from baseline to 10 m for quiet rest (rho = −0.263, *p* = 0.026) and moderate (rho = −0.323, *p* = 0.006) sessions, showing that higher depressive symptoms were associated with a stronger anxiolytic response. Only the moderate session relationship at 10 m was significant after Bonferroni correction (Supplementary Table S1).

When included in the VAS model, BDI was significantly associated with VAS scores (*p* < 0.01), but the significance of the overall interaction and main effects did not change. No Spearman’s rho correlation coefficients were significant between BDI and changes in VAS from baseline to immediately post, 10, or 30 min for any of the sessions (Supplementary Table S2).

### 3.5. Hypothesis #3: prescribed versus preferred exercise

#### 3.5.1. STAI-Y1 results

When the preferred exercise condition was added to the STAI-Y1 model, there were significant main effects of time (*F*_(1.39,31.96)_ = 39.359, *p* < 0.001, ε = 0.695) and session (*F*_(4,92)=_2.779, *p* = 0.03, ε = 0.787), but no significant session by time interaction (*F*_(4.76,109.43)_ = 1.701, *p* = 0.14, ε = 0.595). Pairwise comparisons showed that moderate intensity exercise significantly reduced state anxiety compared to preferred at 30 m (*t* = 2.20, *g* = 0.43, *p* = 0.04) prior to Bonferroni correction. No sessions were significantly different than each other after Bonferroni correction.

#### 3.5.2. Anxiety VAS results

When the preferred exercise condition was added to the VAS model, there was a main effect of time (*F*_(1.89,39.63)_ = 25.533, *p* < 0.001, ε = 0.629), indicating general improvement of anxiety ratings over time, but there was no significant session by time interaction (*F*_(5.12,107.48)_ = 1.274, *p* = 0.28, ε = 0.427) or main effect of session (*F*_(2.95,61.94)_ = 0.368, *p* = 0.77, ε = 0.737). No pairwise comparisons including preferred intensity were significant.

## 4. Discussion

This study explored the influence of light, moderate, and hard prescribed steady-state exercise sessions on state anxiety responses in 24 women with MDD. Moderate intensity exercise significantly reduced state anxiety to a greater extent than quiet rest at 10- and 30-min post-exercise and, while statistically nonsignificant, improvements at other exercise intensities (light, hard) compared to quiet rest were also observed (STAI: Hedges’ *g* = 0.32–0.51, VAS: *g* = −0.025–0.31, all *p*_adj_ > 0.05). While current depressive symptom severity was significantly associated with state anxiety, it did not significantly influence the anxiolytic response to acute exercise. Lastly, preferred intensity sessions were not better at reducing state anxiety than quiet rest and may have been less effective than moderate exercise. This study does not support a linear dose–response relationship between exercise intensity and state anxiety reduction and suggests that moderate prescribed exercise may be the most effective exercise prescription for state anxiety reductions in MDD.

**Figure 1:**
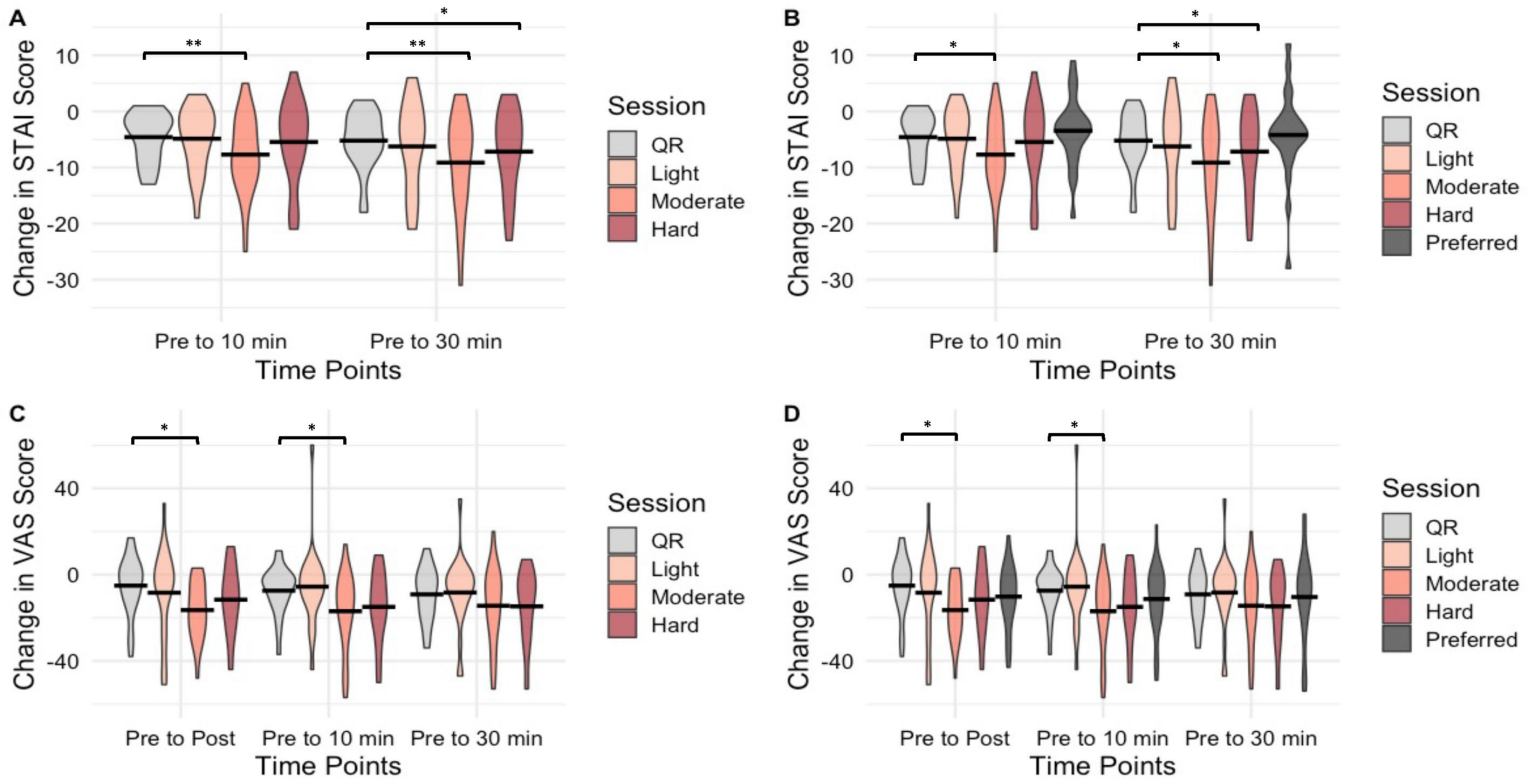
Violin plots of changes in STAI-Y1 and Anxiety VAS scores before and after 30 min of exercise by session and time. **(A,C)** shows STAI-Y1 and Anxiety VAS change scores, respectively, for Aim 1. **(B,D)** shows STAI-Y1 and Anxiety VAS change scores, respectively, for Aim 3, which includes the preferred session. ^*^Indicates statistical significance (*p* < 0.05) of between-group pairwise comparison before Bonferroni adjustment. ^**^Indicates statistical significance (*p* < 0.05) of between-group pairwise comparison after Bonferroni adjustment. STAI-Y1, State–Trait Anxiety Inventory (State version only); VAS, Anxiety Visual Analog Scale; QR, Quiet Rest.

While we hypothesized that state anxiety may increase immediately after hard exercise and then drop below pre-exercise levels at 10- and 30-min post-exercise based on past research in healthy adults [e.g., (32)], we did not find evidence of this (Figure 1). Rather, there was a slightly lower immediate anxiety reduction following hard exercise compared to the other intensities. Although state anxiety decreased over time, the effect was only different from quiet rest following the moderate session with the STAI-Y1 at both 10- and 30-min post-exercise following correction for multiple comparisons, supporting our hypothesis that moderate exercise would be the most effective in reducing state anxiety. The lack of an immediate increase in anxiety in the hard session could be due to a stronger general anxiolytic effect of exercise in women with MDD than has been seen in past research or could have been influenced by external factors. Blanchard et al. (33) found unfit adults experienced psychological distress following hard exercise, and Broman-Fulks et al. (13) found repeated aerobic exercise decreased anxiety sensitivity, both supporting the idea that a more fit/active sample may be less prone to anxious feelings following exercise. Our sample reported being regularly active (77.1 ± 82.9 mean minutes/day of self-reported MVPA). Therefore, the present sample may have been adapted to the physiological responses to hard exercise through interoceptive exposure, thereby being able to interpret higher intensity exercise with a positive, anxiolytic response (34). Moderation of the acute anxiolytic effect of exercise by fitness may also help to explain varying previous findings at hard intensities (10, 13) and favors lower intensity exercise in an untrained population. Considering that the general adult population with depression is likely to be less active than non-depressed counterparts (35) and low fitness is associated with depression (36), moderate intensity exercise may be particularly useful for short-term alleviation of anxiety symptoms in MDD and may be a useful general recommendation for acute psychological benefits across populations.

Although the present data showed a significant decrease in STAI-Y1 scores for moderate exercise compared to quiet rest, no significant effects were seen at any intensity compared to quiet rest using the VAS after Bonferroni correction. However, similar small to moderate effects were found using both measurements at a moderate intensity compared to quiet rest from post to 30 m (*g* = 0.30–0.61). Higher variability in the VAS may have limited power to identify significant differences, so interpretations should be made with caution. Nevertheless, due to its simplicity and quick administration, the VAS added the unique time point immediately post-exercise when a longer set of questionnaires was not expedient. Overall, the STAI-Y1 and VAS both showed moderate exercise to have the largest anxiolytic effects compared to quiet rest.

We hypothesized that anxiolytic effects would strengthen over time, although this was not found. Instead, we saw that effects occurred quickly and then persisted, particularly when performed at a moderate or hard intensity, with reductions falling below clinically significant STAI scores at 30 m post-exercise for both intensities (29). This suggests a durability of the anxiolytic effects likely lasting at least 30 min post-exercise in females with MDD. Our findings are consistent with those of Herring et al. (37), who found that 30 min of exercise resulted in a decrease in state anxiety at 10 m post-exercise in young women with probable GAD (*d* = 0.44), and are larger than Hallgren et al. (38) who found small improvements (*d* = 0.17) 30 min after brief exercise in adults with substance use disorder. In the present study, although there was a decrease in state anxiety following quiet rest at both 10 m and 30 m for both measures, the magnitude of the changes following moderate and hard exercise surpassed the effects of resting quietly indicating a specific anxiolytic response to exercise. Research that evaluates the length of this response beyond 30 min and the response to alternative exercise modes [e.g., high-intensity interval exercise; see (39)] is needed.

Neither considering the severity of depressive symptoms nor having participants choose their intensity meaningfully influenced the state anxiety response to exercise. While Blumenthal et al. (11) found that baseline anxiety levels may inhibit the antidepressant response to exercise training over time, our results (aim #2) suggest that current depression severity did not interfere with the anxiolytic response to acute exercise. It is plausible that participating in the present low-commitment, supervised exercise study resulted in overcoming depression as a perceived barrier to exercise (40) leading to a consistent anxiolytic effect, regardless of depression severity. Although we hypothesized that the preferred intensity session would elicit stronger anxiolytic effects than any prescribed exercise (aim #3), this was not the case and did not largely alter our results. Interestingly, before Bonferroni correction, prescribed moderate exercise was more effective than the preferred session at reducing state anxiety (*g* = 0.43 at 30 m), even though the preferred session was at a moderate intensity according to RPE and HR. This suggests self-selecting exercise intensity was not as beneficial for reducing state anxiety as hypothesized. Prior research suggests that adults with depression are slower to make decisions than otherwise healthy adults (41), which may explain the stronger response to moderate intensity exercise when provided with a specific prescription, rather than having to make the decision oneself. Further, after the end of the final data collection on the fifth visit, participants were shown their personal data and allowed to ask any questions of the research staff. During this period, some participants commented on their low self-efficacy in making the decision on how hard to exercise during the preferred condition which may partially explain the blunted anxiolytic response from the preferred session. Together, the present research underlines prescribed acute exercise’s potential utility in alleviating state anxiety in MDD, regardless of depression severity.

### 4.1. Strengths and limitations

This secondary analysis allowed for the evaluation of the dose–response relationship between exercise intensity and state anxiety. Our study design included identical procedures for all sessions, which allowed for a rigorous comparison between each exercise intensity and quiet rest, but led to smaller effects between groups than uncontrolled pre-post designs, as quiet rest alone had an anxiolytic effect. The between-subjects study design and small sample size also mean the present analysis may have been underpowered to detect meaningful anxiolytic differences between intensities, across time, and based on depression severity. Still, this analysis of the acute effects of exercise intensity on state anxiety provides a novel insight of dose-specific exercise prescription, and as repeated exposure to acute anxiety symptom relief following exercise may provide longer-term reductions in anxiety symptoms, the present results may also inform future exercise training trials focused on treating anxiety symptoms, particularly in those with comorbid depression and anxiety. These findings also suggest that RPE is an effective exercise prescription method, expanding options beyond objective measurements, and may be particularly useful for people with potentially differing perceived and physiological exercise intensities (e.g., people with depression). On average, our sample self-reported higher levels of daily physical activity than may be expected by adults with depression, which may limit the generalizability of these results. Additionally, there was no requirement of pre-existing anxiety or a separate anxious-only group, though participants generally had clinically relevant levels of state anxiety pre-exercise. Future studies evaluating the role of anxiety in a depressed population could benefit from comparisons made to a clinically anxious sample and a healthy control group.

## 5. Conclusion

Women with MDD showed the greatest reduction in state anxiety following moderate intensity steady-state exercise (using an RPE prescription), with reductions persisting at least 30 min. Higher intensity exercise may also be useful in reducing state anxiety in women with MDD, though research investigating potential moderators of the anxiolytic response to hard exercise (e.g., fitness, experience) may prove fruitful. Although self-selecting exercise intensity provides an opportunity for greater autonomy, prescribing moderate exercise appears to be the most consistent method of reducing state anxiety symptoms in women with MDD. Current depression severity may not need to be considered when using exercise for anxiety symptom management in adults with depression, making acute exercise a broadly useful anxiety management tool in this population.

## Data availability statement

The raw data supporting the conclusions of this article will be made available by the authors, without undue reservation.

## Ethics statement

The studies involving human participants were reviewed and approved by the Institutional Review Board at the University of Wisconsin–Madison. The patients/participants provided their written informed consent to participate in this study.

## Author contributions

JM and DC contributed to the study design and data collection. SP and JM completed the statistical analyses. MH provided critical expertise on exercise and anxiety. SP wrote the first draft of the manuscript. All authors contributed to revising, finalizing, and approving the final version of the manuscript.

## Funding

This project was funded, in part, by the Virginia Horne Henry Gift Fund, the University of Wisconsin-Madison Graduate School and the Wisconsin Center for Education Research. None of the funding sources were involved in the study design, collection, analysis, or interpretation of the data.

## Conflict of interest

The authors declare that the research was conducted in the absence of any commercial or financial relationships that could be construed as a potential conflict of interest.

The reviewer CR declared a past co-authorship with one of the authors MH to the handling editor.

## Publisher’s note

All claims expressed in this article are solely those of the authors and do not necessarily represent those of their affiliated organizations, or those of the publisher, the editors and the reviewers. Any product that may be evaluated in this article, or claim that may be made by its manufacturer, is not guaranteed or endorsed by the publisher.
