# Supplementary material for: Dose-response effects of acute exercise intensity on state anxiety among women with depression
Source: Front Psychiatry. 2023 May 12;14:1090077. doi: 10.3389/fpsyt.2023.1090077 (PMC10213268; doi:10.3389/fpsyt.2023.1090077)
Supplement: Supplementary file 1 [file Table_1.docx]

Supplementary Material

| **Supplementary Table 1.** Spearman’s rho correlation coefficients of STAI-Y1 change scores across exercise sessions and BDI-II | | | | |
| --- | --- | --- | --- | --- |
|  | **Spearman’s Rho**  **Pre to 10m** | | **Spearman’s Rho**  **Pre to 30m** | |
| **Session** | **Rho** | ***p*** | **Rho** | ***p*** |
| Quiet Rest | ***-0.263** | ***0.0259** | -0.176 | 0.140 |
| Light | -0.00964 | 0.936 | 0.110 | 0.357 |
| Moderate | ***-0.323** | ***0.00566** | -0.203 | 0.087 |
| Hard | -0.127 | 0.288 | 0.125 | 0.296 |
| Note. * indicates statistical significance at p < 0.05. Abbreviations: BDI-II = Beck Depression Inventory; STAI-Y1 = State-Trait Anxiety Inventory (State version only). | | | | |
