# Supplementary material for: Dose-response effects of acute exercise intensity on state anxiety among women with depression
Source: Front Psychiatry. 2023 May 12;14:1090077. doi: 10.3389/fpsyt.2023.1090077 (PMC10213268; doi:10.3389/fpsyt.2023.1090077)
Supplement: Supplementary file 2 [file Table_2.docx]

Supplementary Material

| **Supplementary Table 2.** Spearman’s rho correlation coefficients of VAS change scores across exercise sessions and BDI-II | | | | | | |
| --- | --- | --- | --- | --- | --- | --- |
|  | **Spearman’s Rho**  **Pre to Immediately Post** | | **Spearman’s Rho**  **Pre to 10m** | | **Spearman’s Rho**  **Pre to 30m** | |
| **Session** | **Rho** | ***p*** | **Rho** | ***p*** | **Rho** | ***p*** |
| Quiet Rest | -0.106 | 0.621 | -0.071 | 0.741 | -0.142 | 0.508 |
| Light | -0.030 | 0.893 | 0.294 | 0.173 | -0.411 | 0.052 |
| Moderate | 0.102 | 0.637 | 0.137 | 0.522 | -0.053 | 0.805 |
| Hard | -0.379 | 0.075 | -0.164 | 0.456 | -0.154 | 0.482 |
| Note. No correlations were statistically significant different from 0 at p < 0.05. BDI-II = Beck Depression Inventory; VAS = Anxiety Visual Analogue Scale. | | | | | | |
